# The Sensory Identity of Caatinga Honeys: From Pollen Profile to Consumer Response

**DOI:** 10.1002/fsn3.72378

**Published:** 2026-10-07

**Authors:** Laís Cabral Madureira de Assis, Eike Guilherme Torres de Souza, Jéssica Moreira de Carvalho, Fabyan Laís de Nascimento Guedes, Katharina Kardinele Barros Sassi, Tatiana Colombo Pimentel, Lary Souza Olegário, Marta Suely Madruga

**Affiliations:** ^1^ Post Graduate Program in Food Science and Technology, Department of Food Engineering, Technology Center Federal University of Paraíba João Pessoa Paraíba Brazil; ^2^ Federal Institute of Paraná Paranavaí Paraná Brazil; ^3^ IPROCAR Research Institute, TECAL Research Group Universidad de Extremadura Cáceres Spain

**Keywords:** botanical origin, Check‐All‐That‐Apply, focus group, honey from *Apis mellifera* bees, sensory wheel

## Abstract

Honey presents distinct sensory characteristics influenced by geographical and botanical origin. This study aimed to characterize the sensory identity of *Apis mellifera* honeys produced in the Caatinga region of Paraíba (Brazil) and to investigate their relationship with consumer perception. Twelve honey samples (mono‐ and polyfloral) were evaluated using a combination of Focus Group, Check‐All‐That‐Apply (CATA), and affective tests. Initially, 551 sensory terms were generated through Focus Group sessions and reduced, through semantic and frequency analyses, to a structured sensory lexicon used to develop a sensory wheel comprising attributes of color, appearance, and aroma. Correspondence analysis revealed two main clusters: light‐colored honeys, associated with sweet, floral, and fruity aromas and botanical sources such as *Croton* spp., *Ziziphus* spp., and *Anadenanthera* spp.; and dark‐colored honeys, mainly from *Mimosa* spp., characterized by woody, toasted, and medicinal notes. Consumer responses indicated positive acceptance and purchase intention for all samples, with higher scores associated with light‐colored honeys exhibiting floral and sweet aromas. In contrast, samples with higher citation frequency showed lower acceptance. The results indicate that sensory characteristics varied among the honey samples associated with different botanical origins, providing insights into the sensory identity of honeys from the Caatinga biome. These findings contribute to product valorization and support strategies for geographical indication and market differentiation.

## Graphical abstract

This study examines the sensory characterization of monofloral and polyfloral honeys produced by *Apis mellifera* bees in the Caatinga region of Paraíba, focusing on their color, appearance, and aroma. Through the application of descriptive methods (Focus Group and CATA), it was possible to generate a Sensory Wheel with terms that can be associated with the botanical origin of the honeys produced in the region, allowing for their identification and distinction through sensory evaluation. Through the conduct of affective tests (Acceptance and Purchase Intention), the main sensory classes and attributes that positively influence consumers' perceptions of this product were identified.

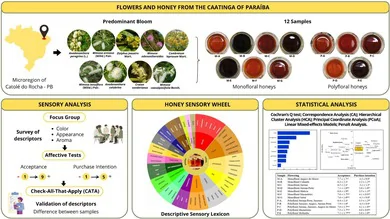

## 1 Introduction

Honey is widely consumed worldwide and is valued for its distinctive sensory properties, which are strongly influenced by both geographical and botanical origin. The diversity of landscapes and plant species across different ecological zones creates favorable conditions for the production of honeys with diverse sensory profiles (Manoko and Mduda 2024).

The northeastern semi‐arid region of Brazil stands out for its environmental conditions that favor beekeeping, including intense natural flowering (De Sousa et al. 2016) and a hot, dry climate. According to data from the 2017 Agricultural Census (IBGE 2023), 94.14% of beekeeping establishments in northeastern Brazil are located in this region. Due to socioeconomic and edaphoclimatic constraints, particularly water scarcity, beekeeping represents an important source of income for local communities.

This region encompasses the Caatinga biome, which is endemic to Brazil and characterized by vegetation adapted to both rainy and dry seasons. These conditions promote continuous nectar and pollen availability throughout the year, with plant species from genera such as *Mimosa* spp., *Croton* spp., and *Ziziphus* spp. (Santos et al. 2015). In addition, environmental stress conditions stimulate increased secondary metabolism in plants, leading to the production of bioactive compounds such as phenolics (Borges and Amorim 2020; Araújo et al. 2023). These factors contribute to the unique identity of honeys produced in this region.

The botanical origin of honey has significant economic relevance, as it directly influences product quality and market value. Light‐colored monofloral honeys are generally more highly valued by consumers, resulting in considerable price variation. Therefore, establishing differentiated quality standards based on botanical origin represents a strategic approach to adding value and supporting geographical indication certification (Carvalho et al. 2026; da Costa, Sousa, Bezerra, et al. 2018; da Costa, Sousa, da Silva, et al. 2018).

Consumer perception studies indicate that the purchase of local foods is often associated with expectations of superior quality and sensory attributes, making sensory characteristics a key factor influencing purchasing decisions (Feldmann and Hamm 2015; Liu et al. 2025).

Descriptive sensory methods play a key role in food characterization by breaking down overall perception into individual sensory attributes (Hofmanová et al. 2020). Among these methods, the Focus Group approach enables the generation of descriptors through guided discussion and interaction among participants (Bertelsen et al. 2021). These descriptors can be organized into sensory wheels, which are effective tools for representing the identity and complexity of food products.

The Check‐All‐That‐Apply (CATA) method is widely used for consumer‐based sensory characterization, allowing participants to select attributes that best describe a product. This approach provides reliable and discriminative data for complex foods such as honey (Jaeger et al. 2020; Gunaratne et al. 2019).

Although there are studies on the descriptive sensory characterization of Brazilian honeys, the literature has prioritized the analysis of honey produced by stingless bees (da Costa, Sousa, Bezerra, et al. 2018; da Costa, Sousa, da Silva, et al. 2018; De Sousa et al. 2016), resulting in a scarcity of research focused on the sensory profile of *Apis mellifera* honey. Furthermore, although well‐established tools exist, such as the odor and aroma wheels developed by Bruneau et al. (2000) and Piana et al. (2004), their application to characterize honeys from other regions is limited since factors such as climate and vegetation confer unique and intrinsic sensory attributes to each production site.

Therefore, this study aimed to characterize the sensory identity of *A. mellifera* honeys produced in the Caatinga region of Paraíba by combining descriptive and consumer‐based sensory approaches, as well as to investigate the relationship between botanical origin and consumer perception.

## 2 Materials and Methods

### 2.1 Samples

Honey from *A. mellifera* bees was collected from beekeepers affiliated with three cooperatives (COOAPIL, COASPA, and COPEMEAF) located in the semi‐arid region of the state of Paraíba, within the Caatinga biome in Brazil. Sampling took place from 2023 to 2024, reflecting the region's climatic conditions, which include rainy seasons (December through June) and dry seasons (July through August). Samples of processed honey were collected after undergoing the procedures of uncapping, centrifugation, and decanting; these samples were placed in transparent plastic bottles and stored at room temperature (28°C ± 2°C) until analysis.

The sampling design was developed to include the main flowering events representative of the Sertão region of Paraíba. Thus, the selection of beekeepers, conducted in partnership with local cooperatives, was based on the following criteria: traceability of floral and geographic origin and the volume produced per flowering event, given that certain flowering events have limited seasonal production. Additionally, the study included samples of monofloral and polyfloral honeys, ensuring the investigation of a wide range of sensory attributes characteristic of the biome.

Although the sample includes the most significant blooms in the region, the selection criteria do not cover all blooms in the Caatinga. However, based on the sample set defined in the study and the methodological design proposed in Section 2.3, it was possible to generate the data needed to establish a representative sensory profile for the honeys produced in this region.

### 2.2 Experimental Design

In total, three experiments were discussed in this article. Focus group for the survey of sensory attributes of appearance, color, and aroma of the 12 honey samples for the development of the sensory wheel of honeys from the Caatinga region of Paraíba. Consumer testing using the Check‐All‐That‐Apply (CATA) to preliminarily confirm the characterization of Caatinga honeys using the descriptors obtained from the Focus Group and identify differences in the perception of attributes for each sample. Application of affective tests focused on evaluating consumer acceptance and purchase intention for each type of honey analyzed.

As the study involved human subjects and biological material, it was previously submitted to and approved by the bioethics committee, CAAE 74712923.4.00005188, and SISGEN, Cadaster n° AAADD65. In all sessions, participants received the Free and Informed Consent Form (TCLE) with information about the method used and its approval by the committee, which was signed by the candidate, agreeing to voluntarily participate in the tests, as required by Resolution n., de 12/12/2012, of the National Health Council.

### 2.3 Methods

#### 2.3.1 Melissopalynological Analysis

To confirm the floral origin of the honey samples, melissopalynological analysis was performed according to the methodology of Barth (1989), with adaptations by Moreira et al. (2023). An aqueous honey solution (0.5 g mL^−1^) was centrifuged at 4000 rpm for 20 min, and allowed to stand for 1 h. The supernatant was removed. Glycerin was added to the precipitate, and the mixture was sealed on a slide with paraffin. At four different points on the slide, 500 pollen grains were counted using an optical microscope (Nikon Optiphot II, 400× and 1000×). The pollen grains were compared with reference images of bee pollen from the University of São Paulo (São Paulo, Brazil). Samples with a pollen representation from a single source greater than 45% were classified as monofloral honeys. The analyses were performed in triplicate for the 12 honeys analyzed in the study. The classification of honeys into monofloral and polyfloral types is described in Table 1.

**TABLE 1:** Geographic description and relative quantity of different pollens in honey from *Apis mellifera* bees from the Caatinga biome located in the Paraíba Semiarid region.

Code	Place of origin^b^	Pollen genero	Pollen species	%	Popular name	Year of collection	Station
*Monofloral honeys* ^a^							
M‐A	São Bentinho	*Anadenanthera* spp. (50.0%)	*Anadenanthera peregrina* (L.) Speg.	50.00%	Angico do Morro	2023	Fall/rainy
			*Mimosa arenosa* (Willd.) Poir.	33.30%	Calumbi		
			Unidentified	16.70%			
M‐B	São Domingos	*Mimosa* spp. (77.2%)	*Mimosa arenosa* (Willd.) Poir.	46.40%	Calumbi	2023	Fall/rainy
			*Mimosa tenuiflora* (Willd.) Poir.	26.20%	Jurema Preta		
			*Ziziphus joazeiro* Mart.	11.00%	Juazeiro		
			*Mimosa arenosa* (Willd.) Poir.	4.60%	Jurema		
M‐C	Catolé do Rocha	*Mimosa* spp. (64.3%)	*Mimosa arenosa* (Willd.) Poir.	51.80%	Jurema	2023	Winter/drought
			*Mimosa ulbrichiana* Harms.	9.30%	*Mimosa ulbrichiana* Harms		
			*Mimosa arenosa* (Willd.) Poir.	3.20%	Calumbi		
			*Combretum leprosum* Mart.	7.70%	Mofumbo		
			*Borreria scabiosoides* Cham. & Schltdl.	2.00%	Cabeça de Velho		
M‐D	Catolé do Rocha	*Mimosa* spp. (70.5%)	*Mimosa tenuiflora* (Willd.) Poir.	70.50%	Jurema Preta	2023	Summer/dry
			*Onagraceae*	14.00%	*Onagraceae*		
			*Ziziphus joazeiro* Mart.	2.80%	Juazeiro		
M‐E	Catolé do Rocha	*Mimosa* spp. (75.4%)	*Mimosa adenantheroides*	65.90%	Malícia	2023	Summer/dry
			*Mimosa tenuiflora* (Willd.) Poir.	9.50%	Jurema preta		
			*Waltheria operculata* Rose.	5.60%	*Waltheria operculata* Rose		
			*Poulsenia armata*	3.60%	Poulsenia armata		
M‐F	Catolé do Rocha	*Croton* spp. (79.2%)	*Croton sonderianus*	79.20%	Marmeleiro	2023	Fall/rainy
M‐G	Matureia	*Mimosa* spp. (96.6%)	*Mimosa caesalpiniifolia* Benth.	96.60%	Sabiá	2023	Fall/rainy
			*Ziziphus joazeiro* Mart.	3.20%	Juazeiro		
			*Croton sonderianus*	0.20%	Marmeleiro		
*Polyfloral honeys*							
P‐A	Catolé do Rocha	*Mimosa* spp. (36.4%)	*Mimosa tenuiflora* (Willd.) Poir.	36.40%	Jurema Preta	2023	Verão/seco
		*Ziziphus* spp. (29.2%)	*Ziziphus joazeiro* Mart.	29.20%	Juazeiro		
P‐B	Catolé do Rocha	*Ziziphus* spp. (37.0%)	*Ziziphus joazeiro* Mart.	37.00%	Juazeiro	2024	Fall/rainy
		*Anadenanthera* spp. (36.0%)	*Anadenanthera colubrina* (Vell.) Brenan	36.00%	Angico		
		*Mimosa* spp. (27.0%)	*Mimosa tenuiflora* (Willd.) Poir.	27.00%	Jurema Preta		
P‐C	Catolé do Rocha	*Mimosa* spp. (44.2%)	*Mimosa arenosa* (Willd.) Poir.	41.20%	Jurema	2023	Winter/drought
		*Ziziphus* spp. (15.1%)	*Ziziphus joazeiro* Mart.	15.10%	Juazeiro		
		*Anadenanthera* spp. (14.6%)	*Anadenanthera peregrina* (L.) Speg.	14.6%	Angico do Morro		
		Unidentified (8.0%)	Unidentified	8.00%			
P‐D	Jericó	*Croton* spp. (30.0%)	*Croton sonderianus*	30.00%	Marmeleiro	2023	Fall/rainy
		Unidentified (10.0%)	Unidentified	10.00%			
P‐E	Catolé do Rocha	*Combretum* spp. (33.3%)	*Combretum leprosum* Mart.	33.30%	Mofumbo	2023	Winter/drought
		Unidentified (10.0%)	Unidentified	10.00%			

*Note:* M‐A: Monofloral Angico do Morro, M‐B: Monofloral Calumbi, M‐C: Monofloral Jurema, M‐D: Monofloral Jurema Preta, M‐E: Monofloral Malícia, M‐F: Monofloral Marmeleiro, M‐G: Monofloral Sabiá, P‐A: Polyfloral Juazeiro, Angico, Jurema Preta, P‐B: Polyfloral Jurema Preta, Juazeiro, P‐C: Polyfloral Jurema, Juazeiro, Angico do Morro, P‐D: Polyfloral Marmeleiro and unidentified pollens, P‐E: Polyfloral Mofumbo and unidentified pollens.

^a^ Monofloral honey: Predominant pollen (> 45%); Polyfloral honey: Secondary pollen (15 a 45%) and important minor pollen (3 a 15%).

^b^ Catoté do Rocha [La (−6.34103), Lo (−37.7496 6°20′28″ South, 37°44′59″ West), Al (272 m)]; Jericó [La (−6.54275), Lo (−37.8056 6°32′34″ South, 37°48′20″ West), Al (250 m)]; Aparecida [La (−6.77168), Lo (−38.0479 6°46′18″ South, 38°2′52″ West), Al (224)]; São Domingos [La (6°50′4″ South), Lo (37°53′9″ West), Al (211 m)]; Matureia [La (07°16′01″ South), Lo (37°21′05″ West), Al (815 m)]; Poço de José de Moura [La (−6.57198), Lo (−38.5294 6°34′19″ South, 38°31′46″ West), Al (285 m)]; Mãe d'água [La (−7.25279), Lo (−37.4328 7°15′10″ South, 37°25′58″ West), Al (420 m)].

#### 2.3.2 Development of the Sensory Lexicon

##### 2.3.2.1 Recruiting the Panel

The panel consisted of 27 untrained participants, including 14 women and 13 men aged 18–65, among whom were professors and technical specialists in the food sector with college degrees, students in the Food Engineering program and the Graduate Program in Food Science and Technology—PPGCTA at the Federal University of Paraíba—UFPB, and honey consumers with college degrees. All participants had prior experience in food sensory analysis.

The meetings took place over in three sessions, each with nine panelists, a mediator who conducted the session, and an assistant who collected and organized the panel's oral contributions. The mediator and assistant were the same for all three sessions, thereby ensuring consistency in explanations, clarification of questions, and guidance during the discussions. The number of participants in each group was established according to Lawless and Heymann (2010) and De Pelsmaeker et al. (2019), who recommend recruiting up to 12 people per session for the conduct of Focus Groups for sensory analysis. The Focus Groups took place in a meeting room in a controlled environment equipped with artificial lighting using neutral white light, where the panelists gathered around a round table. The mediator ensured everyone's participation, keeping the discussion focused on obtaining the descriptors in a clear manner and answering any questions that arose.

##### 2.3.2.2 Preparation and Presentation of Honeys

The honey samples were presented in transparent, cylindrical, odorless glass containers and coded with a three‐digit random number, with identical samples coded identically for all panelists. Twenty grams of each honey sample were served for sensory evaluation at a temperature of 20°C ± 1°C. The 12 honeys were divided, at random, into three groups of four samples, which were presented sequentially to the evaluators until all groups had been fully analyzed. The samples in each block, such as M1, M2, M3, and M4, were presented simultaneously and equally to all evaluators and analyzed from left to right until completion. For each focus group session, the same sample blocks were used, but the order in which the blocks were presented and the arrangement of the samples varied. Once prepared, the samples were evaluated within 24 h.

##### 2.3.2.3 Sample Evaluation Protocol

Before analyzing the honeys, the panelists were briefed on the focus group's purpose and on how to proceed. They then received the samples, a predefined color scale based on the honeys being analyzed, and a list of aroma and appearance descriptors to aid in identification, though this did not restrict the use of other terms not included in the list. The list of terms was generated using sensory descriptors for honey found in the literature (Piana et al. 2004; Mongi and Ruhembe 2024), which were adapted to represent the sample under study. Meanwhile, the color scale was created by evaluating the actual colors of the samples and generating shades on the RGB (Red, Green, and Blue) scale that matched and encompassed the sample set.

For the aroma evaluation, transparent plastic spoons were provided so panelists could stir the honey, thereby encouraging the release of volatile compounds. To record the descriptors, each participant received a form and a pen. Between sample analyses, the panelists were instructed to reset their sense of smell; coffee beans were provided to neutralize aromas, along with crackers and water, with a 1‐min break between each sample analyzed. A 5‐min break was allowed between each group of four samples evaluated, ensuring standardization and minimizing panelist fatigue. Each focus group session lasted an average of 1 h and 30 min. It is important to note that the panel of evaluators was unaware of the floral origin of the honeys being analyzed to ensure there was no interference during the evaluation.

##### 2.3.2.4 Generations of Terms

Initially, the descriptive sensory terms were collected through individual evaluations by the panelists, in which the characteristics of the honey samples were described based on the sensory parameter being analyzed (color, appearance, and aroma), and similarities and differences between the samples were identified.

After the complete analysis of each honey, a discussion coordinated by the mediator took place, where the terms listed by each panelist were addressed, and the perceptions described by the panel were observed, with the possibility of reviewing the samples again to facilitate the identification and removal of terms mentioned or the addition of new terms. The discussion was held for all samples, thus ending the session.

#### 2.3.3 Data Consolidation, Term Selection, and Development of the Sensory Wheel

After the completion of the three focus group sessions, the lists of descriptors generated by each group were compiled and compared to assess consistency among them. To reach consensus on the terms generated in each session, selection and exclusion criteria for descriptors were applied until the final terms for constructing the sensory wheel were defined.

A total of 551 raw descriptors were generated across the three focus group sessions, with an average of 184 ± 12 terms per session. Similarity was observed among the sensory descriptors generated, even across different groups, indicating the methodological standardization that was implemented and the evaluative quality of the panel.

The refinement and selection of sensory descriptors were carried out in two main stages. Stage 1 consisted of semantic filtering based on De Pelsmaeker et al. (2019), organized into five criteria: (1) grouping of repetitive terms or synonyms (e.g., sweet/sweetish; freshness/refreshing; twig/bark/wood), retaining the most frequently cited term; (2) removal of semantic opposites (e.g., homogeneous/heterogeneous; translucent/opaque); (3) elimination of hedonic or emotional descriptors (e.g., good, bad, apathetic); (4) removal of quantitative intensity terms (e.g., intense, mild, strong, weak); and (5) removal of terms irrelevant to the honey matrix (e.g., smell of a river, glue, rubber) (Xia et al. 2025). This initial filtering reduced the number of terms from 551 to 154.

Next, Stage 2 was conducted, based on the citation frequency criterion according to Koch et al. (2012). In this stage, the number of mentions for each of the 154 terms was tallied across the three focus group sessions, selecting only those descriptors that achieved a mention frequency of 5% or higher in at least one of the analyzed samples. This stage ensured the retention of attributes relevant to the characterization of regional honeys, resulting in a final reduction from 154 to 63 sensory descriptors suitable for composing the lexicon and the sensory wheel.

Of these 63 terms, 55 were organized in the outermost layer of the wheel, composed of attributes with greater specificity; 8 were allocated to the second layer and added to 11 more terms, totaling 19, representing more comprehensive attributes that integrate the specific ones. The innermost layer, on the other hand, consists of only two attributes that correspond to the analyzed classes “aroma” and “visual” (representing color and appearance).

The terms in the second layer of the sensory wheel categorize specific descriptors in a sectorized manner. Thus, the additional terms were added to provide more specific descriptors that do not detract from the general term commonly cited in the focus group sessions. Therefore, the 11 additional descriptors maintain the meaning and contemplate the descriptive characteristics of the honeys analyzed.

#### 2.3.4 Consumer Test

The CATA test was conducted in accordance with Ares and Jaeger (2023) to obtain consumer responses and to preliminarily confirm the characterization of Caatinga honeys using the sensory terms generated in the Focus Group. For a comprehensive analysis of the 12 honeys, the judges had to hold two sessions, evaluating six honeys in each, during which all participants evaluated all the samples.

The CATA questionnaire provided to consumers was structured into four main sections. The first section presented the Informed Consent Form (ICF), containing information about the study, the professionals involved, the subject of the study, and the criteria for participation in the study: being a honey consumer and having no allergies or health problems related to honey consumption. The second and third sections addressed, respectively, the sociodemographic profile and consumption profile of the evaluators, as detailed in Table 2. Finally, the fourth section corresponded to the sample evaluation stage. Thus, to compose the CATA questionnaire, the 17 general aroma terms and the 11 specific visual terms, covering color and appearance, were selected, totaling 28 terms.

**TABLE 2:** Profile of participants in the descriptive characterization of samples using the CATA method and affective tests of acceptance and purchase intention.

Consumer profile	Σ	%
Number of participants (*n*)	100	100%
*Gender*		
Male	49	49%
Female	50	50%
I'd rather not say	1	1%
*Age group*		
Between 18 and 24 years old	57	57%
Between 25 and 34 years old	25	25%
Between 35 and 44 years old	10	10%
Between 45 and 54 years old	5	5%
Between 55 and 64 years old	0	0%
65 years or older	3	3%
*Education level*		
Elementary school	1	1%
High school complete	13	13%
Technical education	2	2%
Higher education incomplete	55	55%
Higher education complete	2	2%
Postgraduate	27	27%
*Monthly income range*		
Less than or equal to one (01) minimum wage	50	50%
Between one (01) and three (03) minimum wages	29	29%
Between three (03) and six (06) minimum wages	13	13%
Between three (06) and six (09) minimum wages	5	5%
More than nine (09) minimum wages	3	3%
*Frequency of honey consumption*		
Never	0	0%
Rarely	27	27%
Monthly	57	57%
Fortnightly	7	7%
Weekly	7	7%
Daily	2	2%

In the test, consumers evaluated the aroma, color, and appearance of the same 12 honey samples used in the Focus Group, in two blocks of six samples, conducted in separate sessions. The samples from block one (samples analyzed in the first session) and block two (samples analyzed in the second session) were selected at random, and the order of presentation followed a Williams Latin square design to mitigate the effects of order, with a random sequence of attributes for each participant using an electronic questionnaire.

The honeys were presented individually and in a randomized order. For the evaluation, 20 g of each sample were served at a temperature of 20°C ± 1°C, accompanied by water, a salted cracker, and coffee beans to cleanse the sense of smell, with a 1‐min interval between the analysis of each sample. A color scale prepared in advance was made available on the workbenches for analyzing the color of the samples. All sensory evaluations were conducted at the Food Sensory Analysis Laboratory of the Federal University of Paraíba, in a controlled environment under the same conditions for all evaluators, with neutral white artificial lighting. This methodological approach aimed to confirm preliminarily the terms selected for the aroma, color, and appearance attributes generated by the Focus Group for honeys from the Caatinga biome of Paraíba, where the evaluators participating in the study were unaware of the botanical origin of the honeys under study. The flavor of the honeys was not evaluated, therefore, the samples were not ingested by the consumers.

#### 2.3.5 Affective Tests

Affective tests of acceptability and purchase intention were conducted for the aroma, color, and appearance of the 12 honeys. To assess consumer acceptability, a 9‐point hedonic scale was used, where 1 = Dislike Extremely and 9 = Like Extremely. The scale was applied to each sensory aspect analyzed (color, aroma, and appearance) and to the overall acceptance of the sample. To assess purchase intention, a 5‐point scale was applied, where 1 = would not buy and 5 = would definitely buy. The tests were conducted in conjunction with the CATA test, using an electronic questionnaire. The taste of the honeys was not evaluated; therefore, consumers did not taste the samples.

#### 2.3.6 Data Analysis

The statistical analysis of the data obtained from the CATA test was performed using complementary univariate and multivariate approaches. Initially, Cochran's *Q* test was applied to each sensory attribute to determine whether its citation frequency differed significantly among the honey samples (*α* = 0.05). Correspondence analysis (CA) was subsequently performed to explore the associations between honey samples and sensory attributes. The input matrix consisted of the sample × sensory‐attribute citation‐frequency matrix obtained from the CATA data, in which each attribute was recorded as a binary response for each consumer.

Hierarchical Cluster Analysis (HCA) was performed using OriginPro software, version 2024, based on the sample × sensory attribute frequency matrix obtained from the CATA test. Euclidean distance was used as the dissimilarity measure, and the average of the groups (average linkage) method was employed for hierarchical clustering. The resulting dendrogram was used to identify groups of honey samples with similar sensory profiles.

Principal Coordinate Analysis (PCoA) was performed using the sample × sensory‐attribute citation‐frequency matrix obtained from the CATA data. Pairwise dissimilarities between samples were calculated using the chi‐square distance, and the resulting dissimilarity matrix was subjected to PCoA. The resulting coordinates were interpreted according to the proportion of total variation represented by each axis and the relative positioning of the honey samples in the ordination space.

Consumer acceptance and purchase‐intention data were analyzed using linear mixed‐effects models to account for the repeated‐measures structure of the experiment, since the same consumers evaluated multiple honey samples. Honey sample was included as a fixed effect and consumer as a random effect. Model assumptions were assessed by examining the distribution of residuals and homogeneity of variance. No missing observations were identified in the dataset. When a significant effect of honey sample was detected, post hoc pairwise comparisons were performed using Tukey's test with adjustment for multiple comparisons and a 95% confidence interval. Statistical significance was established at *α* = 0.05. The CATA, CA, PCA, acceptability, and purchase intention analyses were performed using XLSTAT software, version 2019.2.2.

Penalty analysis was performed using XLSTAT 2022.2.1.1299 based on the binary CATA responses and overall acceptance scores. For each attribute, respondents were classified according to the presence or absence of the attribute, and mean overall acceptance scores were calculated for both groups. The mean impact was calculated as the difference between the mean acceptance score when the attribute was present and when it was absent. Positive values indicated a positive association of the attribute with overall acceptance, whereas negative values indicated a negative association. Statistical significance was assessed using the statistical procedure implemented in XLSTAT, with *p* < 0.05 considered significant. A 20% frequency threshold was used as the reference criterion in the mean‐impact‐versus‐frequency plot.

## 3 Results and Discussion

Among the 12 samples, seven were classified as monofloral (Angico do Morro, Calumbi, Jurema, Jurema Preta, Malícia, Marmeleiro, and Sabiá), while five were polyfloral, composed of combinations of these botanical sources.

### 3.1 Development of the Sensory Lexicon

For the description of sensory characteristics, 12 honey samples with distinct sensory profiles were considered in order to obtain as many descriptive attributes as possible. The selected terms resulted in a consolidated set of specific attributes, as detailed in Table 3, subsequently organized into a coherent category system, presented in the form of a sensory wheel, according to the description by Asih et al. (2021) for the Focus Group discussion and professional study of the sensory wheel of aroma, color, and appearance.

**TABLE 3:** Descriptive terms with a frequency of > 5% for at least one of the honeys from *Apis mellifera* bees from the Caatinga biome, evaluated and generated during focus group sessions.

Category	Attribute	M‐A	M‐B	M‐C	M‐D	M‐E	M‐F	M‐G	P‐A	P‐B	P‐C	P‐D	P‐E
*Visual*													
Color	Light amber	48%	0%	15%	0%	0%	59%	11%	48%	0%	0%	67%	0%
	Amber	22%	0%	26%	0%	0%	41%	63%	48%	4%	19%	33%	0%
	Dark amber	4%	0%	56%	33%	15%	0%	26%	0%	81%	78%	0%	78%
	Light brown	0%	11%	0%	30%	48%	0%	0%	0%	0%	0%	0%	22%
	Reddish brown	0%	41%	0%	0%	0%	0%	0%	0%	0%	0%	0%	0%
	Dark brown	0%	44%	0%	0%	0%	0%	0%	0%	0%	0%	0%	0%
*Visual*													
Apperance	Shiny	78%	70%	85%	81%	89%	96%	93%	85%	93%	89%	81%	81%
	Translucent	85%	44%	85%	67%	74%	96%	85%	96%	81%	89%	100%	85%
	Viscous	96%	89%	93%	89%	100%	96%	96%	89%	85%	93%	100%	100%
	Homogeneous	70%	63%	70%	56%	63%	67%	70%	63%	70%	63%	70%	67%
	Consistent	41%	100%	52%	89%	59%	56%	63%	59%	93%	56%	52%	44%
*Aroma*													
Bee	Bee	< 5%	< 5%	< 5%	7%	< 5%	< 5%	< 5%	< 5%	< 5%	< 5%	< 5%	< 5%
	Beeswax	< 5%	7%	< 5%	< 5%	< 5%	< 5%	< 5%	< 5%	< 5%	< 5%	< 5%	< 5%
	Honeycomb	11%	< 5%	< 5%	7%	11%	7%	7%	7%	< 5%	< 5%	< 5%	< 5%
	Honey	41%	48%	22%	52%	52%	48%	81%	52%	11%	59%	41%	63%
	Propolis	< 5%	22%	< 5%	< 5%	19%	11%	22%	< 5%	< 5%	< 5%	< 5%	11%
Sweetness	White sugar	< 5%	7%	< 5%	< 5%	< 5%	11%	< 5%	< 5%	< 5%	< 5%	< 5%	< 5%
	Sweetness	33%	7%	< 5%	52%	44%	33%	41%	48%	7%	19%	37%	22%
	Glucose syrup	< 5%	< 5%	< 5%	< 5%	< 5%	15%	< 5%	< 5%	7%	< 5%	< 5%	< 5%
Woody	Wood	19%	26%	15%	41%	19%	< 5%	< 5%	< 5%	33%	< 5%	7%	26%
Animal	Leather	< 5%	< 5%	30%	< 5%	< 5%	< 5%	< 5%	< 5%	< 5%	< 5%	< 5%	< 5%
	Sulfur	< 5%	< 5%	11%	< 5%	< 5%	< 5%	< 5%	< 5%	< 5%	< 5%	< 5%	< 5%
	Sweat	< 5%	< 5%	< 5%	< 5%	< 5%	< 5%	< 5%	< 5%	< 5%	< 5%	< 5%	15%
	Urine	< 5%	< 5%	19%	< 5%	< 5%	< 5%	< 5%	< 5%	< 5%	< 5%	< 5%	< 5%
Sugarcane	Brown sugar	7%	26%	< 5%	< 5%	11%	< 5%	7%	< 5%	< 5%	< 5%	11%	< 5%
	Sugarcane juice	11%	7%	< 5%	< 5%	< 5%	< 5%	< 5%	< 5%	< 5%	< 5%	< 5%	< 5%
	Molasses	19%	26%	< 5%	< 5%	19%	< 5%	< 5%	< 5%	< 5%	< 5%	< 5%	< 5%
	Jaggery	< 5%	26%	< 5%	< 5%	< 5%	< 5%	7%	< 5%	< 5%	7%	11%	22%
Caramel	Burnt sugar	< 5%	< 5%	7%	11%	< 5%	< 5%	< 5%	< 5%	< 5%	11%	< 5%	11%
	Caramel syrup	< 5%	< 5%	< 5%	< 5%	7%	7%	< 5%	< 5%	< 5%	< 5%	< 5%	< 5%
	Caramel	22%	7%	< 5%	30%	< 5%	< 5%	< 5%	15%	< 5%	< 5%	< 5%	< 5%
Spices	Vanila	7%	< 5%	< 5%	< 5%	7%	< 5%	< 5%	< 5%	< 5%	7%	< 5%	< 5%
	Cinnamon	< 5%	< 5%	< 5%	7%	< 5%	< 5%	< 5%	26%	< 5%	19%	< 5%	< 5%
	Clove	7%	< 5%	< 5%	< 5%	< 5%	< 5%	< 5%	11%	< 5%	7%	15%	< 5%
	Spices	11%	< 5%	< 5%	19%	11%	< 5%	11%	11%	< 5%	< 5%	22%	< 5%
	Ginger	11%	7%	< 5%	< 5%	< 5%	< 5%	< 5%	11%	< 5%	7%	< 5%	< 5%
	Spicy	< 5%	15%	11%	< 5%	< 5%	< 5%	< 5%	< 5%	< 5%	< 5%	7%	< 5%
	Pungent	< 5%	< 5%	7%	< 5%	< 5%	< 5%	< 5%	< 5%	< 5%	< 5%	< 5%	< 5%
Citrus fruit	Citrus	7%	< 5%	< 5%	19%	11%	15%	30%	7%	7%	11%	19%	11%
Fermented	Acid	< 5%	< 5%	7%	< 5%	< 5%	< 5%	< 5%	< 5%	22%	< 5%	< 5%	< 5%
	Beer	< 5%	< 5%	< 5%	7%	< 5%	< 5%	< 5%	< 5%	< 5%	< 5%	< 5%	< 5%
	Fermented	< 5%	7%	11%	< 5%	15%	< 5%	< 5%	< 5%	< 5%	< 5%	< 5%	7%
	Mold	< 5%	< 5%	22%	< 5%	< 5%	< 5%	< 5%	< 5%	26%	< 5%	< 5%	< 5%
Floral	Flowers	7%	11%	< 5%	11%	37%	22%	41%	37%	< 5%	33%	15%	7%
Fresh fruit	Pineapple	< 5%	< 5%	< 5%	< 5%	7%	< 5%	< 5%	< 5%	< 5%	< 5%	< 5%	15%
	Cashew	< 5%	< 5%	< 5%	< 5%	< 5%	< 5%	< 5%	< 5%	< 5%	< 5%	< 5%	7%
	Fruity	37%	7%	< 5%	< 5%	26%	15%	33%	11%	< 5%	26%	19%	7%
Dry fruit	Dry fruit	< 5%	30%	< 5%	< 5%	< 5%	< 5%	< 5%	< 5%	< 5%	< 5%	< 5%	< 5%
	Nuts	7%	< 5%	< 5%	< 5%	< 5%	< 5%	< 5%	< 5%	< 5%	< 5%	< 5%	< 5%
Smoke	Smoky	< 5%	< 5%	52%	< 5%	7%	< 5%	< 5%	< 5%	41%	< 5%	15%	< 5%
	Burnt	< 5%	7%	19%	< 5%	< 5%	< 5%	< 5%	< 5%	30%	< 5%	< 5%	7%
Fresh herbaceous	Eucalyptus	< 5%	< 5%	< 5%	7%	< 5%	< 5%	< 5%	< 5%	< 5%	< 5%	< 5%	< 5%
	Fresh herbaceous	19%	< 5%	< 5%	< 5%	< 5%	< 5%	< 5%	< 5%	< 5%	7%	< 5%	< 5%
	Menthol	7%	< 5%	< 5%	< 5%	< 5%	7%	< 5%	7%	< 5%	< 5%	< 5%	< 5%
	Refreshing	19%	15%	< 5%	26%	11%	7%	19%	33%	< 5%	7%	44%	< 5%
Dry herbaceous	Tea	7%	< 5%	< 5%	< 5%	< 5%	< 5%	11%	< 5%	< 5%	11%	< 5%	< 5%
	Leaf	< 5%	< 5%	11%	7%	< 5%	< 5%	< 5%	< 5%	26%	< 5%	< 5%	< 5%
	Tobacco	< 5%	< 5%	< 5%	15%	< 5%	< 5%	< 5%	< 5%	< 5%	< 5%	< 5%	< 5%
Medicinal	Honey cough syrup	< 5%	41%	< 5%	7%	7%	7%	19%	11%	< 5%	11%	< 5%	11%
	Medice	< 5%	< 5%	7%	7%	7%	< 5%	< 5%	< 5%	7%	< 5%	< 5%	< 5%
Toasted	Roast	< 5%	< 5%	< 5%	< 5%	7%	7%	< 5%	< 5%	15%	< 5%	< 5%	< 5%
	Coffee	7%	15%	15%	< 5%	7%	< 5%	< 5%	< 5%	19%	< 5%	< 5%	< 5%
	Toasted	< 5%	7%	11%	< 5%	< 5%	< 5%	< 5%	< 5%	< 5%	< 5%	< 5%	< 5%

*Note:* M‐A: Monofloral Angico do Morro, M‐B: Monofloral Calumbi, M‐C: Monofloral Jurema, M‐D: Monofloral Jurema Preta, M‐E: Monofloral Malícia, M‐F: Monofloral Marmeleiro, M‐G: Monofloral Sabiá, P‐A: Polyfloral Juazeiro, Angico, Jurema Preta, P‐B: Polyfloral Jurema Preta, Juazeiro, P‐C: Polyfloral Jurema, Juazeiro, Angico do Morro, P‐D: Polyfloral Marmeleiro and unidentified pollens, P‐E: Polyfloral Mofumbo and unidentified pollens.

The sensory wheel for Caatinga honeys was generated, containing three hierarchical levels, with 55, 19, and 2 descriptive terms, respectively, from the outermost layer to the innermost, as shown in Figure 1. The outermost level contains the specific attributes that were grouped into secondary descriptors in the second (intermediate) level. The innermost level consists of the sensory dimensions of “aroma” and “visual.”

**FIGURE 1:**
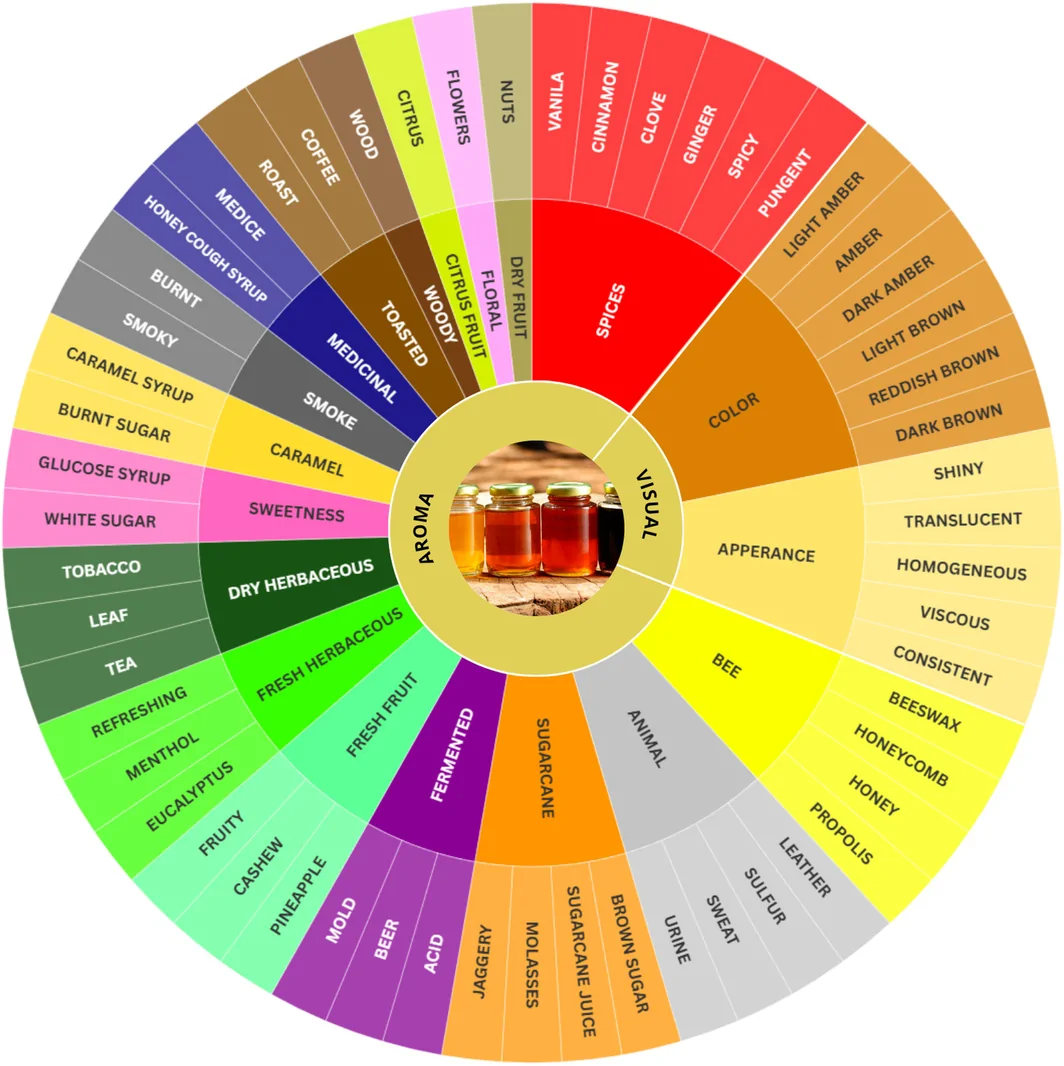
Sensory wheel of honey from the semi‐arid region of Paraíba.

The sensory wheel of honey from the Caatinga biome characterizes this matrix as bright, translucent, homogeneous, and viscous, with colors ranging from light amber to dark brown. The aromatic bouquet of honeys from this region consists of notes such as “sugary,” represented by descriptors such as sweet, caramel, sugarcane, and candy, and “floral and fruity,” with terms such as flowers, fresh fruits, citrus fruits, dried fruits, and spices. More distinctive terms, such as wood, dry herbaceous, and fresh herbaceous, were also listed in the characterization. It is worth noting that off‐flavors were identified, which may be associated with “defects,” such as terms like smoke, fermented, animal, roasted, and medicinal. One possible explanation for why these smoky, roasted, and fermented aromas are attributed to honey is the use of hives containing Africanized bees in the study, which require the use of smokers during honey collection, and by environmental conditions with high relative humidity (around 70%) that can favor fermentation, despite the region's low rainfall. According to Arnaud et al. (2008), the management practices applied can also influence honey aroma, with the potential to interfere with the generation of negative terms for this sample when not properly performed.

As can be seen in the first wheel of odors and aromas for honey, created by Bruneau et al. (2000), which brings together attributes and terms used to describe European monofloral honeys, consisting of seven descriptors (floral/fresh fruit, fresh, chemical, woody, vegetal, spoiled, warm) in the innermost layer, followed by 19 (nineteen) terms in the middle layer and 64 (sixty‐four) terms in the outer layer, with positive and negative terms similar to those attributed to the samples in this study.

In addition to environmental conditions, good management practices, and collection methods, other factors can affect the sensory characteristics of honey and, consequently, the generation of descriptive terms, with pollen composition being one of the most important. The sensory wheel (Figure 1) presents all the attributes described for honeys from the Caatinga biome, located in the semiarid region of Paraíba, whether monofloral or polyfloral. By evaluating the botanical origin and composition of pollens, it is possible to verify the specific sensory characterization of these types (Figure 2).

**FIGURE 2:**
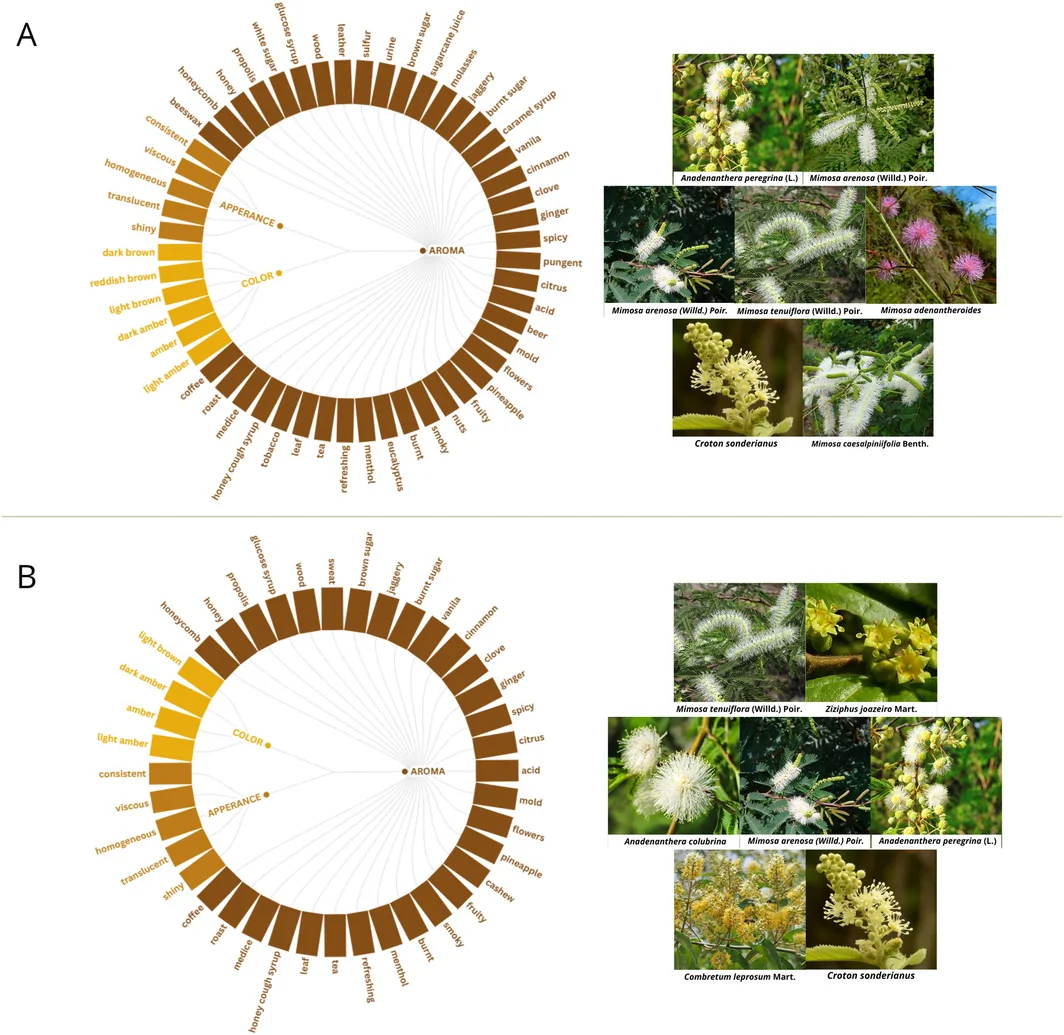
Flowers and descriptors of monofloral (A) and polyfloral (B) honeys from the Caatinga biome, located in the semi‐arid region of Paraíba.

Monofloral honeys presented a greater number of aroma terms (42 descriptors), 35 of which were positive (e.g., white sugar and caramel syrup) and 7 negative (e.g., beer and sulfur). The predominance of a floral origin gives honey unique and well‐defined characteristics (Carvalho et al. 2026), enabling the identification of a wider range of specific attributes that make up its aromatic profile. However, polyfloral honeys, characterized by the variability of their pollen content, have a less distinct profile (31 aroma descriptors) resulting from contributions from different flowers, which interferes with the delimitation of specific characteristics and impacts the perception and more detailed identification of this typology.

It is worth mentioning that twenty‐nine (29) aroma descriptors were common to both monofloral and polyfloral honeys, although thirteen (13) descriptors were listed only for monofloral honeys, namely: wax, white sugar, sugarcane juice, molasses, leather, sulfur, urine, caramel syrup, pungent, beer, nuts, eucalyptus, and tobacco. Polyfloral honeys had two aroma descriptors, sweat and cashew, not listed for monofloral honeys.

Despite the difference in the number of specific descriptors, both classifications, monofloral and polyfloral, have the same secondary terms, such as floral, herbaceous, and medicinal, which were also observed in the study conducted by da Costa, Sousa, Bezerra, et al. (2018) and da Costa, Sousa, da Silva, et al. (2018) with honeys from the same region, reinforcing the influence of geographical location on the sensory characteristics of honey.

In addition to aroma, color differentiation was evident: polyfloral honeys showed a narrower range, from light amber to brown, while monofloral honeys covered the six colors described in the wheel. Appearance was the only attribute analyzed that did not differ in relation to the number of terms identified for the two types of honey.

The sensory wheel developed in this study should be regarded as a descriptive and exploratory tool, as it provides a structured representation of the sensory descriptors for honey produced by *A. mellifera* in the Caatinga biome. The descriptors generated and organized through the focus group were subsequently evaluated using the CATA method, and the frequency with which they were mentioned in the honey samples analyzed provided support for the relevance and applicability of the proposed sensory vocabulary. However, further studies are needed, using independent samples and batches from different harvests, botanical profiles and production regions, as well as trained sensory panels, to assess the tool's wider applicability.

### 3.2 Check‐All‐That‐Apply (CATA)

According to the sensory attributes previously defined by the Focus Group, the consumer test was conducted using the CATA method, presenting significant differences for the descriptive characterization of the honeys analyzed, as shown in Table 4. This table includes the 17 aroma terms described in the second layer of the sensory wheel, as well as specific descriptors for color (6 terms) and appearance (5 terms).

**TABLE 4:** Citation frequency (%) of sensory terms of the studied honeys and multiple pair comparison analysis (Sheskin) and Cochran's *Q* test (*p*‐values) for the Check‐All‐That‐Apply (CATA) test with monofloral and polyfloral honeys from the Caatinga biome, located in the semiarid region of Paraíba.

Attributes	M‐A	M‐B	M‐C	M‐D	M‐E	M‐F	M‐G	P‐A	P‐B	P‐C	P‐D	P‐E	*p*
*Apperance*													
Shiny	100^b^	91^a^	97^ab^	97^ab^	95^ab^	100^b^	98^b^	100^b^	100^b^	99^b^	100^b^	97^ab^	**< 0.001**
Translucent	100^c^	62^a^	99^c^	88^bc^	77^b^	100^c^	99^c^	99^c^	99^c^	95^c^	100^c^	96^c^	**0.000**
Viscous	95^a^	98^a^	94^a^	100^a^	95^a^	91^a^	95^a^	95^a^	93^a^	97^a^	93^a^	91^a^	0.142
Homogeneous	100^a^	99^a^	99^a^	96^a^	100^a^	98^a^	98^a^	98^a^	99^a^	97^a^	100^a^	98^a^	0.397
Consistent	96^ab^	95^ab^	91^ab^	99^b^	93^ab^	83^a^	87^ab^	94^ab^	89^ab^	89^ab^	92^ab^	84^a^	**< 0.001**
*Color*													
Light amber	31^b^	0^a^	8^a^	0^a^	0^a^	60^cd^	38^b^	0^a^	42^bc^	3^a^	69^d^	0^a^	**0.000**
Amber	56^e^	0^a^	31^bcd^	2^a^	0^a^	35^cd^	40^cde^	12^ab^	46^de^	20^abc^	28^bcd^	1^a^	**0.000**
Dark amber	9^ab^	0^a^	33^cd^	8^ab^	1^a^	4^ab^	21^bc^	66^e^	12^ab^	46^d^	1^a^	6^ab^	**0.000**
Light brown	4^ab^	6^ab^	28^c^	24^c^	13^abc^	1^a^	1^a^	21^bc^	0^a^	29^c^	2^a^	66^d^	**0.000**
Reddish brown	0^a^	14^ab^	0^a^	46^d^	29^c^	0^a^	0^a^	1^a^	0^a^	2^a^	0^a^	25^bc^	**0.000**
Dark brown	0^a^	80^d^	0^a^	20^b^	57^c^	0^a^	0^a^	0^a^	0^a^	0^a^	0^a^	2^a^	**0.000**
*Aroma*													
Sweetness	55^bcde^	41^abc^	32^ab^	58^cde^	46^abcde^	69^e^	68^de^	30^a^	68^de^	55^bcde^	59^cde^	45^abcd^	**< 0.001**
Woody	12^abc^	31^d^	26^bcd^	32^d^	28^cd^	12^abc^	9^ab^	31^d^	10^abc^	18^abcd^	6^a^	20^abcd^	**< 0.001**
Animal	3^ab^	4^ab^	13^bc^	5^ab^	10^abc^	1^a^	3^ab^	16^c^	0^a^	1^a^	3^ab^	5^ab^	**< 0.001**
Caramel	29^ab^	32^ab^	23^ab^	19^a^	29^ab^	26^ab^	30^ab^	23^ab^	26^ab^	43^b^	28^ab^	33^ab^	**0.046**
Spices	14^a^	13^a^	18^a^	21^a^	10^a^	22^a^	16^a^	9^a^	11^a^	17^a^	12^a^	12^a^	0.135
Fermented	1^a^	11^abc^	6^abc^	5^abc^	9^abc^	1^a^	4^abc^	15^bc^	3^ab^	5^abc^	5^abc^	16^c^	**< 0.001**
Floral	36^cd^	9^a^	15^ab^	27^abcd^	16^abc^	34^bcd^	39^d^	15^ab^	28^abcd^	23^abcd^	38^d^	17^abc^	**< 0.001**
Citrus fruit	19^ab^	4^a^	5^a^	7^a^	10^a^	13^ab^	19^ab^	5^a^	18^ab^	6^a^	26^b^	10^a^	**< 0.001**
Fresh fruit	16^b^	1^a^	7^ab^	6^ab^	4^ab^	10^ab^	13^ab^	6^ab^	14^ab^	5^ab^	8^ab^	11^ab^	**0.003**
Dry fruit	11^a^	10^a^	9^a^	17^a^	10^a^	12^a^	8^a^	16^a^	7^a^	8^a^	15^a^	13^a^	0.367
Smoke	2^a^	17^ab^	50^c^	6^a^	13^a^	4^a^	7^a^	32^b^	2^a^	7^a^	3^a^	7^a^	**0.000**
Fresh herbaceous	18^ab^	9^ab^	3^a^	19^ab^	12^ab^	20^b^	12^ab^	8^ab^	18^ab^	11^ab^	16^ab^	11^ab^	**0.007**
Dry herbaceous	11^a^	17^ab^	22^ab^	17^ab^	17^ab^	15^ab^	9^a^	30^b^	9^a^	14^ab^	13^ab^	11^a^	**0.001**
Medicinal	30^a^	31^a^	17^a^	19^a^	28^a^	22^a^	29^a^	17^a^	32^a^	24^a^	25^a^	31^a^	0.081
Bee	60^bcd^	49^abc^	34^a^	59^bcd^	70^cd^	59^bcd^	70^cd^	37^ab^	76^d^	65^cd^	57^abcd^	58^bcd^	**< 0.001**
Sugarcane	40^abcd^	76^e^	34^ab^	59^cde^	62^de^	40^abcd^	31^ab^	24^a^	36^abc^	47^abcd^	38^abc^	48^bcd^	**< 0.001**
Toasted	4^a^	37^d^	34^d^	26^cd^	27^cd^	7^ab^	6^a^	24^bcd^	4^a^	12^abc^	2^a^	14^abc^	**0.000**

*Note:* M‐A: Monofloral Angico do Morro, M‐B: Monofloral Calumbi, M‐C: Monofloral Jurema, M‐D: Monofloral Jurema Preta, M‐E: Monofloral Malícia, M‐F: Monofloral Marmeleiro, M‐G: Monofloral Sabiá, *p*‐A: Polyfloral Juazeiro, Angico, Jurema Preta, *p*‐B: Polyfloral Jurema Preta, Juazeiro, *p*‐C: Polyfloral Jurema, Juazeiro, Angico do Morro, *p*‐D: Polyfloral Marmeleiro and unidentified pollens, *p*‐E: Polyfloral Mofumbo and unidentified pollens. Different letters on the same line indicate a significant difference between the samples (Q‐Cochran's Test, *p* < 0.05). The bold values correspond to *p*‐values < 0.001, indicating a statistically significant difference in a sensory attribute between samples.

In total, there were 148 participants, 100 of whom completed both test sessions. Consequently, only the responses from the 100 participants who evaluated the complete set of 12 samples were considered, and incomplete data were discarded.

Analyzing the proportions (%) of CATA terms among the sensory attribute classes, color is the only one that presents terms with a significant difference (*p*‐value < 0.05) for all samples. In contrast, the appearance and aroma classes show a distinct behavior, with samples not differing from each other for some attributes. However, significant effects were observed across the three classes analyzed, indicating that the method detected differences in consumer perception of the sensory characteristics of honeys from the Caatinga biome.

Regarding color characteristics, all attributes were mentioned and were predominant in at least one honey. Among the samples, the one that obtained the highest percentage of mentions for a single color was M‐B (honey from *Mimosa* spp.—calumbi flowering), with 80% for dark brown, while the greatest dispersion of citations among the attributes was for sample M‐C (honey from *Mimosa* spp.—jurema flowering), where the highest percentage (33%) was for dark amber. Samples M‐D (honey from *Mimosa* spp.—black jurema flowering) and P‐E (honey from *Combretum* spp.—mofumbo flowering) had the highest percentage of citations for reddish brown (46%) and light brown (66%), respectively, being the only honeys with a higher proportion (%) for these colors. The lighter shades of the color scale, light amber and amber, were cited with a higher percentage for samples P‐D (69%) and M‐A (56%), honey from *Croton* spp.—marmeleiro flowering and honey from *Anadenanthera* spp.—angico do morro flowering, in that order.

Based on pollen type, the colors were organized into well‐defined groups according to floral composition. It is worth noting that polyfloral honeys, despite not having a predominance of pollen (≤ 45%), have the characteristics of the combination that forms their composition, with the pollen with the highest percentage significantly influencing the sensory characteristics, or even the combination of pollens in proportion influencing these characteristics more significantly (Roldán et al. 2011).

The M‐F and P‐D samples were evaluated with light amber coloring, being the only honeys with the genus *Croton* spp. (quince blossom) in their composition. The amber color was observed in three samples, M‐A, M‐G, and P‐B, which are monofloral honeys of *Anadenanthera* ssp. (angico do morro flowering) and *Mimosa* spp. (sabiá flowering) as their predominant genera, respectively, while polyfloral honey contains both of these genera.

Samples M‐C, P‐A, and P‐C belong to the genus *Mimosa* spp. (Jurema and black jurema flowers) in higher percentages and were described with a higher number of citations for dark amber color, while honeys M‐B and M‐E, also of the genus *Mimosa* spp. (calumbi and malícia flowers), were classified as dark brown. The colors light brown and reddish brown were described for samples P‐E, which have the pollen genus *Combretum* spp. (mofumbo flowering) in the highest percentage, and sample M‐D, monofloral honey from *Mimosa* spp. (black jurema flowering).

Thus, it is possible to see that honeys with the same pollen genus in their composition were grouped. However, a genus is a taxonomic category that groups biologically related species, so different species within the same genus can exhibit distinct sensory characteristics, as observed with *Mimosa* spp. honeys, where not all were classified as a single color.

Additionally, eight of the twelve honeys evaluated (M‐A, M‐C, M‐F, M‐G, P‐A, P‐B, P‐C, P‐D) were described as amber in color, ranging from light amber to dark amber. In contrast, for darker colors, from light brown to dark brown, only four honeys (M‐B, M‐D, M‐E, P‐E) had a higher percentage of citations. One of the main factors contributing to color variation in this matrix is its derivation from different plant sources, due to the presence of pigments such as carotenoids and flavonoids (Terrab et al. 2003; Al‐Farsi et al. 2018). This coloration can range from almost colorless to dark brown, a variation associated with floral diversity.

Thus, honeys with a predominance of pollen, monofloral honeys, had a greater variety of colors. In contrast, polyfloral honeys were restricted to shades of amber, with the exception of sample P‐E, described as light brown. This behavior can be explained by the fact that monofloral honeys have well‐defined colors depending on their predominant flowering, whereas a greater variety of colors is observed in multifloral honeys. Meanwhile, polyfloral honeys, a combination of different blooms, result in a limited range of lighter tones due to the mix of pollens that give them varied colors. It is worth emphasizing that honeys of the genus *Croton* spp. (quince blossom) were described as having a lighter color, that is, light amber, while those of the genus *Mimosa* spp. (calumbi and malícia blossoms) were described as dark brown. Similar results were obtained by Carvalho et al. (2026) for monofloral honeys from the Caatinga of Paraíba of the genera *Croton* spp. and *Mimosa* spp., which were classified according to the Pfund scale as “extra light amber” and “dark amber,” respectively.

Regarding visual characteristics, the color of honey is also related to appearance. As for brightness, it is possible to perceive its relationship with translucency and coloration, where honeys that presented a percentage equal to 100% for the attributes “bright” and “translucent” were the same ones that presented lighter colors (M‐A amber, M‐F, and P‐D light amber, blooms of the genera *Anadenanthera* spp. and *Croton* spp.) according to consumer perception. Regarding the perception of light passing through the samples, the M‐B and M‐E honeys (*Mimosa* spp., calumbi, and malícia flowers) were described as less translucent, a behavior distinct from that of the other samples, which did not differ from each other. This result can be explained by the darker color of these honeys, which were classified as dark brown, with the highest percentages at 80% and 57%, respectively.

According to the evaluators' perception, all honeys, regardless of flowering or pollen composition, are viscous and homogeneous, with little differentiation, indicating similar rheological characteristics among honeys from the Caatinga biome. From a rheological point of view, honey is a material with a variable molecular structure due to the presence of compounds such as sugars, acids, proteins, small amounts of minerals, pigments, phenolic compounds, and colloids, among others (Smanalieva and Senge 2009; Wei et al. 2010; Witczak et al. 2011; Carvalho et al. 2026). The ratio of fructose to glucose and its relationship with moisture are factors that determine the crystallization rate and, as a result, are responsible for a large part of the rheological properties of honey (Lazaridou et al. 2004; Oroian et al. 2013; Špánik 2014; Witczak et al. 2011; Dobre et al. 2012; Carvalho et al. 2026). These factors directly impact the sensory characteristics of this matrix.

Sugars, which are the main components of honey, depend mainly on botanical and geographical origins, and to a lesser extent on factors such as climate, processing, and storage conditions (Carvalho et al. 2026; Dobre et al. 2012; Ouchemoukh et al. 2010), which explains the behavior found, where there was no significant difference between the honeys analyzed in terms of viscosity and homogeneity, as also evidenced by Escuredo et al. (2014) in their study, where strong correlations were found between the rheological properties of the honeys and their places of origin in Spain and Romania.

On the other hand, the consistency of the samples—penetration capacity—was perceived differently, with M‐D honey (genus *Mimosa* spp., black jurema blossom) being described as the most consistent honey, while M‐F and P‐E honeys (genus *Croton* spp., marmeleiro—flowering and genus *Combretum* spp., mofumbo flowering) were the least dense/consistent, with the other honeys presenting intermediate descriptions in this appearance attribute. In the study by Oroian et al. (2013), the measurement, prediction, and correlation of several physical properties of honey were conducted, and it was proposed that surface tension (consistency) may be related to fluidity. Thus, the data obtained indicate that consumers perceived a relationship between these two properties, with the “viscous” attribute not differing between samples; however, the highest percentage of citation (100%) was observed for sample M‐D, while the lowest (84%) was observed for honey P‐E.

The observed differences in perceived consistency may be associated with variations in sugar composition, particularly the fructose‐to‐glucose ratio, as well as moisture content among the samples. Honeys with higher glucose content tend to exhibit greater crystallization and apparent viscosity, while higher fructose levels are associated with more fluid characteristics. Additionally, botanical origin may influence these parameters. In the study conducted by Carvalho et al. (2026), honeys from the genera *Croton* spp., *Anadenanthera* spp., and *Mimosa* spp. were classified as having a slow or zero propensity for crystallization; however, other intrinsic factors such as water activity and soluble solids content differed among the genera. Honeys from the genus *Croton* spp. showed higher water activity and lower soluble solids content compared to honeys from *Anadenanthera* spp. and *Mimosa* spp., corroborating the sensory perception of these honeys by the panelists.

Analyzing the characteristic aroma attributes, the descriptors “bee,” referring to the specific terms honey, honeycomb, propolis, and wax, and “sweet” were, in general, the most representative, with high percentages in the description of the 12 honeys analyzed. The range of variation for the term “bee” was 34% to 76%, while the “sweet” aroma varied between 30% and 69%, followed by the attribute “sugar cane,” related to the aromas of molasses, brown sugar, and raw sugar, which obtained a frequency of 24%–76% for the samples. These results were not surprising, given that honey has a “sweet” sensory characteristic.

The descriptors perceived by a smaller number of evaluators were “animal” with only 16% for the highest percentage of citations, being the only attribute that obtained 0% for one of the 12 honeys analyzed, and “fermented,” with a range of variation between 1% and 16%, followed by the aromas of “fresh fruit” and “dried fruit,” which varied from 1% to 16% and 7% to 17%, respectively.

It can also be observed that the terms “spices,” “dried fruit,” and “medicinal” were applied to all honeys, but did not show a statistically significant difference between them (*p* > 0.05), thus being attributes that characterize the honeys produced in the caatinga biome, but which are not capable of promoting distinction between the different blooms.

Studies have identified numerous characteristic volatile compounds in honey samples, demonstrating their importance as essential odorants (Špánik 2014). Among the existing range are aroma‐active compounds that generate the impression of odors similar to beeswax, honey, citrus, fresh, floral, pungent, green, herbal, fruity, sulfur, coffee, roasted, sweaty, vinegar, moldy, clove, vanilla, among others (Mahmoud et al. 2024), which vary according to the type of honey.

Terms identical and/or similar to those used in the present study for the sensory characterization of honey were described in the odor and aroma wheel developed by Piana et al. (2004) for European monofloral honeys, among which are: woody, fresh, floral, fresh fruit, vegetable, spoiled, sweetness, freshness, and acidity. In addition to similar terms, the aroma wheel of Piana et al. (2004) consists of distinct descriptors such as bitter, chemical, warm, and astringent. Therefore, it is possible to identify terms commonly associated with the analyzed matrix, as well as unique properties that characterize the different origins.

In an integrated manner, the analysis of different descriptive classes is a useful tool for explaining the behavior and grouping of honeys based on their sensory characteristics of color, appearance, and aroma, as shown in Figure 3A. Using the projective map of correspondence analysis, 73.25% of the relationships between samples and attributes are explained, with components F1 and F2 accounting for 46.47% and 26.78% of the respective variabilities.

**FIGURE 3:**
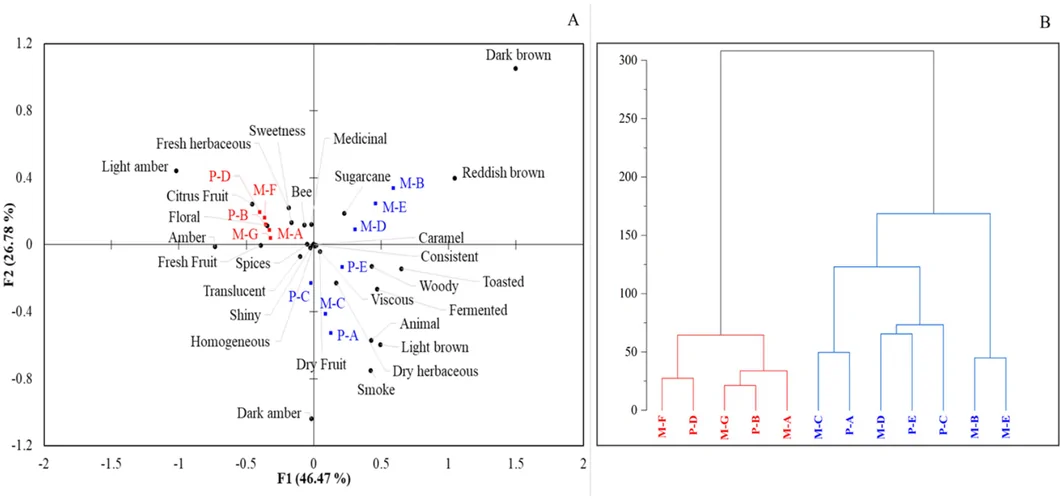
Correspondence analysis chart (A) and hierarchical cluster analysis (B) between monofloral and polyfloral honeys from the Caatinga biome, located in the Semi‐arid region of Paraíba, and the sensory attributes analyzed in the Check‐All‐That‐Apply (CATA) test. M‐A: Monofloral Angico do Morro, M‐B: Monofloral Calumbi, M‐C: Monofloral Jurema, M‐D: Monofloral Jurema Preta, M‐E: Monofloral Malícia, M‐F: Monofloral Marmeleiro, M‐G: Monofloral Sabiá, P‐A: Polyfloral Juazeiro, Angico, Jurema Preta, P‐B: Polyfloral Jurema Preta, Juazeiro, P‐C: Polyfloral Jurema, Juazeiro, Angico do Morro, P‐D: Polyfloral Marmeleiro and unidentified pollens, P‐E: Polyfloral Mofumbo and unidentified pollens.

Along the F1 component, which explains the greatest variability in the data, two clusters can be observed. On the positive side of F1, there was a grouping of darker‐colored honeys (light brown, reddish brown, and dark brown) with the aromas “woody, smoky, animal, sugarcane, toasted, and dry herbaceous.” Except for sample P‐E (*Combretum* spp.), these are all of the genus *Mimosa* spp., with M‐B being Calumbi blossom, M‐C being Jurema blossom, M‐D being black Jurema blossom, M‐E being Malícia blossom, and P‐A being black Jurema and Juazeiro blossoms.

In contrast, honeys with a lighter color, such as light amber and amber, were correlated with the aromas “sweet, floral, fruity (citrus and fresh fruits) and bee,” grouping on the negative side of F1, with a predominance of honeys of the genus *Crotron* spp., M‐F and P‐D, both from quince blossoms, and *Anadenanthera* spp. (M‐A angico do morro flora, M‐G sabiá blossoms, and P‐B juazeiro, angico do morro, and *Mimosa*). These clusters were not formed randomly. As shown in Figure 3B, the hierarchical cluster analysis corroborates the results from PCoA (Figure 3A), revealing the formation of groups based on pollen composition. These findings reinforce the association between botanical origin and the sensory attributes of honey, although it is acknowledged that these attributes may also be influenced by other factors, such as storage, harvesting, maturation, and processing conditions, as well as climatic variability.

The floral origin and volatile compound content of plant nectar are directly related to the aroma properties of honey (Li et al. 2024). Thus, several studies have combined sensory and chemical analyses to investigate volatile/aroma/odor‐active compounds in honey (Seisonen et al. 2015; Tahir et al. 2016; Siegmund et al. 2017; Kortesniemi et al. 2018; Li et al. 2024). In addition to the relationship between color and aroma, the appearance characteristics, despite being concentrated in the center of the graph indicating little influence on the grouping of samples, are divided into the principal component axes, where on the positive side of F1 are the terms viscous and consistent, attributed mainly to samples with dark colors and a more intense woody aroma, toasted, and sugarcane, while on the negative side are characteristics that refer to brightness, translucency, and homogeneity, which are attributes more representative of light honeys with a floral, sweet, and fruity aroma.

Mongi and Ruhembe (2024) in honeys of different floral and geographical origins, where samples described as having greater color intensity (darker) were associated with more intense aromas and greater viscosity, while samples with greater whiteness intensity (lighter) were associated with clarity. These results show that sensory characteristics can distinguish the origin of honeys, especially when there is an association between different properties related to color, appearance, and aroma classes. These properties are among the essential qualities that influence consumer decision‐making in selecting, purchasing, and consuming honey (Ciappini et al. 2016).

#### 3.2.1 Penalty Analysis

To investigate the influence of the presence and absence of CATA attributes on preference scores, a penalty analysis was conducted. Figure 4A shows the graph of the average impact on preference, highlighting the significant attributes (*p* < 0.05). The results indicate that eight CATA descriptors significantly and positively influenced consumers' average preference for the honeys. It is also important to note that, despite the presence of off‐flavors in the sensory profile of the region's honeys, these attributes are not sufficiently perceptible to negatively influence consumer preference.

**FIGURE 4:**
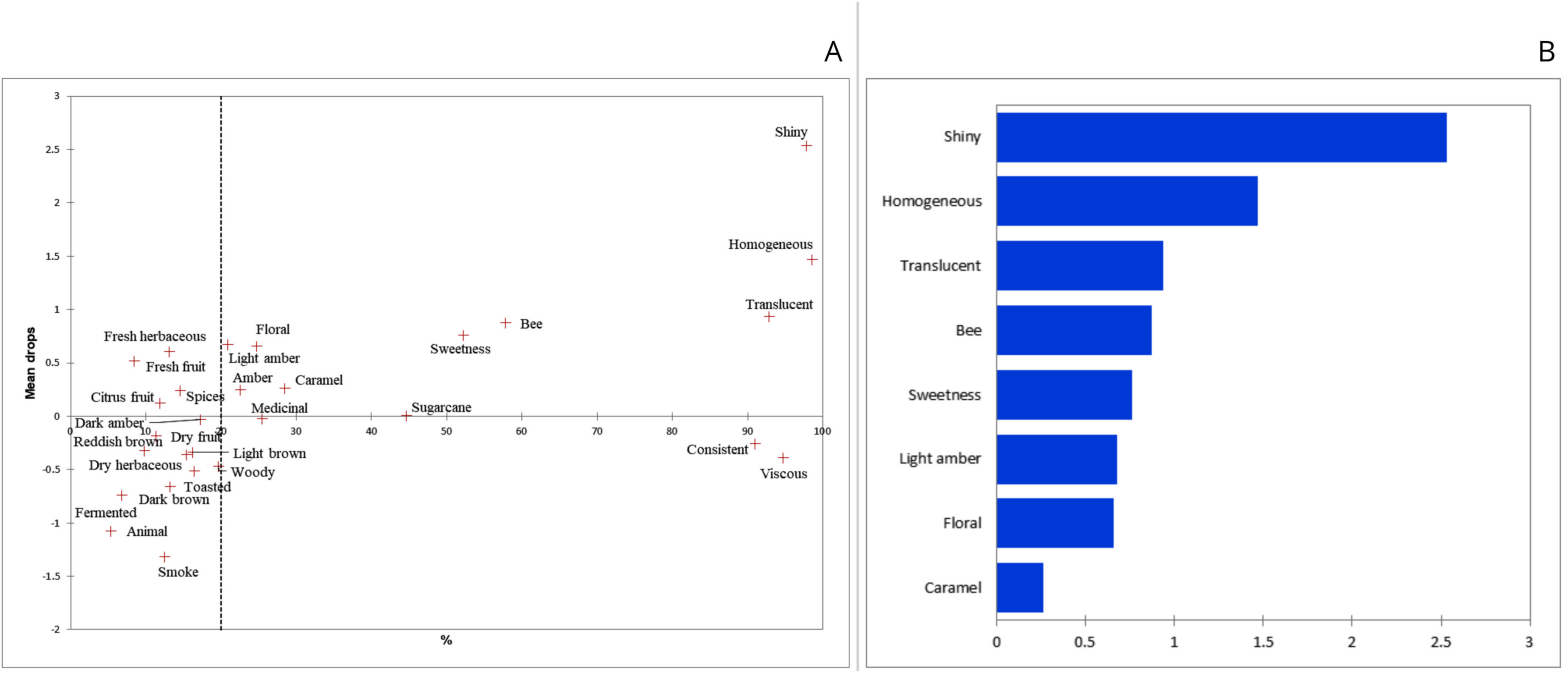
Penalty analysis of sensory attributes (A) and identification of drivers of liking (B) that impact acceptability.

As shown in the scatter plot in Figure 4B, descriptors such as animal, smoke, and fermented have the lowest frequency of mention, the higher citation frequency, and consequently the least impact on the sensory evaluation. Meanwhile, the attributes located in the upper right quadrant of the graph, such as the appearance descriptors shiny, homogeneous, and translucent, and the aroma descriptors honey, sweet, and floral, define the main sensory profile of the honeys and are widely perceived by consumers due to their high intensity.

### 3.3 Affective Tests

The affective tests for acceptance and purchase intention were administered in conjunction with the CATA test, resulting in a total of 100 complete responses regarding the evaluation of the 12 samples. As shown in Table 5, the consumer acceptance scores range from 6.3 ± 2.1 to 7.9 ± 1.6 on a 9‐point scale.

**TABLE 5:** Means of acceptance^1^ and purchase intention^2^ levels for 12 samples of monofloral and polyfloral honeys from the Caatinga biome, located in the semiarid region of Paraíba.

Sample	Flowering	Acceptance	Purchase intention
M‐A	Monofloral Angico do Morro	7.7 ± 1.7^cd^	4.2 ± 0.9^d^
M‐B	Monofloral Calumbi	6.3 ± 2.1^a^	3.2 ± 1.2^ab^
M‐C	Monofloral Jurema	6.5 ± 2.1^a^	3.1 ± 1.3^a^
M‐D	Monofloral Jurema Preta	7.1 ± 1.8^abc^	3.6 ± 1.0^bc^
M‐E	Monofloral Malícia	6.8 ± 1.9^ab^	3.5 ± 1.1^ab^
M‐F	Monofloral Marmeleiro	7.6 ± 1.7^bcd^	4.1 ± 0.9^cd^
M‐G	Monofloral Sabiá	7.6 ± 1.6^bcd^	4.1 ± 0.8^d^
P‐A	Polyfloral Jurema Preta, Juazeiro	6.4 ± 2.1^a^	3.3 ± 1.2^ab^
P‐B	Polyfloral Juazeiro, Angico, Jurema Preta	7.9 ± 1.6^d^	4.3 ± 0.8^d^
P‐C	Polyfloral Jurema, Juazeiro, Angico do Morro	7.6 ± 1.7^bcd^	4.1 ± 1.0^cd^
P‐D	Polyfloral Marmeleiro	7.5 ± 1.7^bcd^	4.0 ± 0.9^cd^
P‐E	Polyfloral Mofumbo	7.1 ± 1.7^abcd^	3.6 ± 1.0^bc^

*Note:* M‐A: Monofloral Angico do Morro, M‐B: Monofloral Calumbi, M‐C: Monofloral Jurema, M‐D: Monofloral Jurema Preta, M‐E: Monofloral Malícia, M‐F: Monofloral Marmeleiro, M‐G: Monofloral Sabiá, P‐A: Polyfloral Juazeiro, Angico, Jurema Preta, P‐B: Polyfloral Jurema Preta, Juazeiro, P‐C: Polyfloral Jurema, Juazeiro, Angico do Morro, P‐D: Polyfloral Marmeleiro and unidentified pollens, P‐E: Polyfloral Mofumbo and unidentified pollens. Different letters in the same column differ statistically by the Tukey test at 5% of significance.

^1^ 9‐point scale (1 = disliked very much and 9 = liked very much).

^2^ 5‐point scale (1 = would not buy and 5 = would definitely buy).

The samples P‐B, M‐A, M‐F, M‐G, P‐C, and P‐D showed no statistical difference and received the best evaluation, while the samples M‐C, M‐B, and P‐A, which also did not differ, obtained lower acceptance. By correlating these data with the sensory characteristics of the honeys, it is possible to determine which attributes are most relevant to acceptance.

The principal coordinate graph (Figure 5) shows the relationship between sensory attributes and consumer acceptance. It can be observed that color class has a more significant influence on acceptability, as the descriptors are located farther from the center of the graph, better defining the groups formed. Looking at the positive side of F1, which explains the greater variability in the terms, we see the colors light amber and amber, suggesting that lighter colors are more acceptable, which is directly correlated with the attributes shiny and translucent, the main appearance characteristics that influence consumer approval. Regarding the aroma class, the main attributes for acceptance were medicinal, caramel, fresh herbaceous, citrus, and fresh fruits, honey, sweet, and floral, also located on the right side of F1.

**FIGURE 5:**
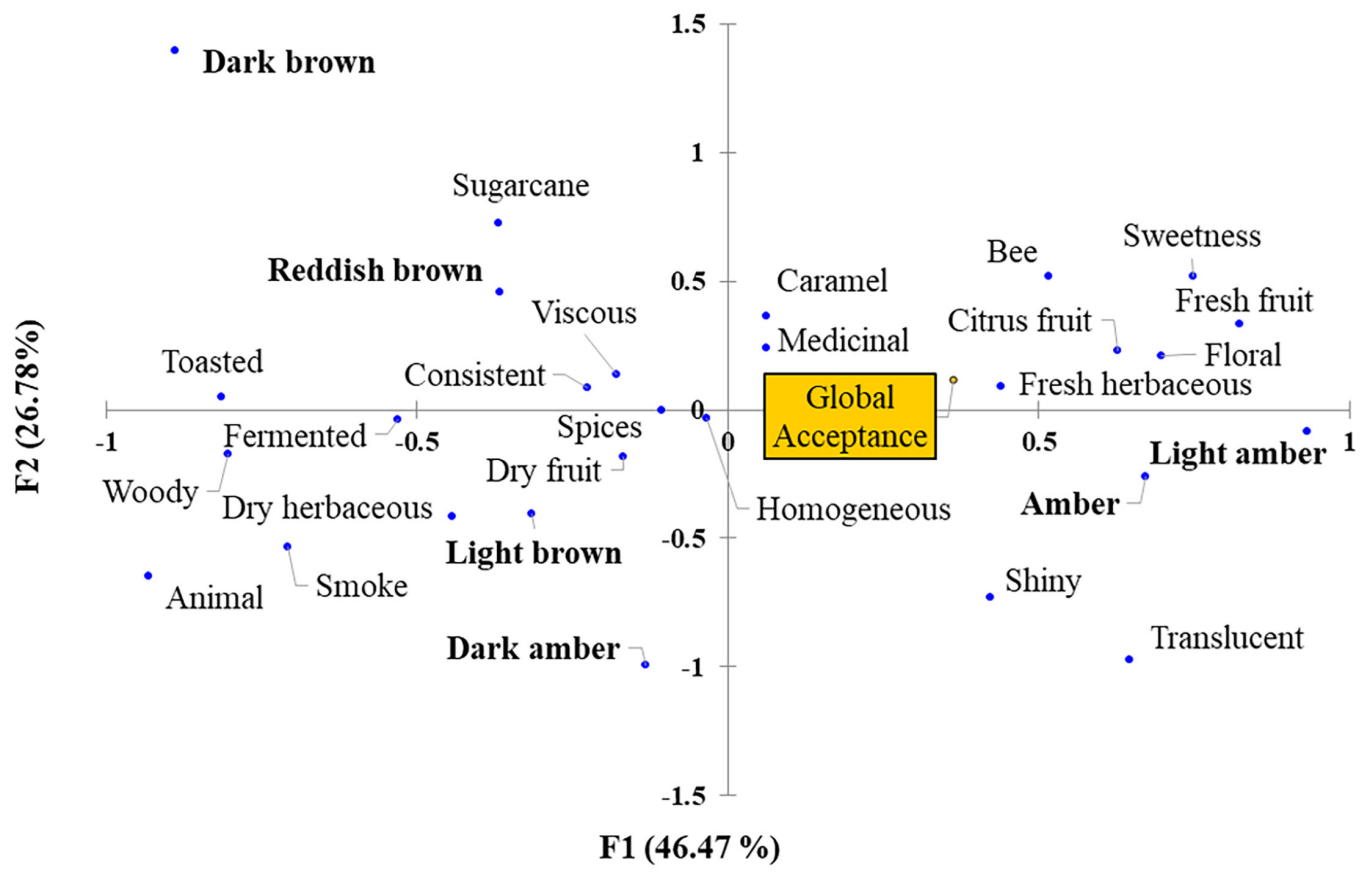
Principal coordinate analysis graph measuring the influence of acceptability on the sensory attributes of monofloral and polyfloral honeys from the Caatinga biome, located in the Semi‐Arid region of Paraíba.

According to the purchase intention data described in Table 5, the polyfloral honeys P‐B, P‐C, and P‐D, and the monofloral honeys M‐A, M‐F, and M‐G were the ones that presented the highest average among consumers, while the sample M‐C, monofloral honey from *Mimosa* spp. (jurema flower) obtained the lowest result.

These data corroborate the acceptance profile generated by the evaluators, highlighting that honeys with a lighter color and traditional honey aroma, as well as floral, citrus, and freshness, have a higher purchase intention. Sample M‐C (jurema), according to Table 3 (focus group data), had the highest percentage for the smoke attribute, as well as high values for the animal and dark amber attributes, which were located on the negative side of F1 and F2 in Figure 5, indicating the lowest acceptance. However, it is important to note that all honeys showed a positive purchase intention, with averages ranging from 3.1 to 4.3 on a 5‐point scale.

Thus, in addition to highlighting which sensory characteristics effectively influence the decision between honey types, the result shows that polyfloral honeys, despite having fewer descriptive attributes according to the methods applied, sometimes do not have the same value compared to monofloral honeys, can have attributes that are equally attractive to consumers, since there was no statistical difference between P‐B, M‐A, and M‐G (juazeiro/angico/jurema, angico do morro, and sabiá blooms).

## 4 Conclusion

This study revealed that the sensory characteristics of *A. mellifera* honeys produced in the Caatinga region of Paraíba were associated with botanical variations and could be described using an integrated sensory approach. The combination of Focus Group, CATA, and affective tests enabled the development of a sensory lexicon and wheel for describing the sensory characteristics observed. Monofloral honeys showed more specific sensory profiles, whereas polyfloral samples exhibited greater complexity. Color and aroma were important drivers of consumer perception, with higher acceptance associated with lighter‐colored honeys presenting sweet, floral, and fruity notes.

These results highlight the potential of sensory characterization to distinguish between honeys from the same region and to support strategies for adding value to the product. Although botanical origin is a key factor, factors such as climatic conditions, harvesting, ripening, processing, and storage also influence the sensory profile. It is also important to note that the sensory profile identified in this study reflects the exploratory associations specific to the set of samples analyzed. Future studies should investigate the relationship between sensory attributes and chemical composition, as well as extend the sampling to other regions, thereby enhancing the characterization of Brazilian honeys.

## Author Contributions

**Laís Cabral Madureira de Assis:** conceptualization, investigation, writing – original draft, visualization, data curation, writing – review and editing. **Eike Guilherme Torres de Souza:** software, formal analysis, conceptualization, writing – original draft. **Jéssica Moreira de Carvalho:** investigation. **Fabyan Laís de Nascimento Guedes:** investigation. **Katharina Kardinele Barros Sassi:** methodology, validation, conceptualization, writing – review and editing. **Tatiana Colombo Pimentel:** formal analysis, software, methodology, writing – review and editing. **Lary Souza Olegário:** software, formal analysis, writing – original draft, supervision, methodology, validation, writing – review and editing, conceptualization. **Marta Suely Madruga:** conceptualization, funding acquisition, writing – original draft, visualization, writing – review and editing, project administration, resources, supervision, data curation, validation.

## Funding

This work was supported by the CNPQ/MCTI/ASSOCIATION A.B.E.L.H.A. (Process number 401168/2022‐0).

## Ethics Statement

As the study involved human subjects and biological material, it was previously submitted to and approved by the ethics committee, CAAE 74712923.4.00005188, and SISGEN, Cadastre n° AAADD65, in research conducted at the Federal University of Paraíba.

## Consent

All authors in this study consent to publish this article and the related data.

## Conflicts of Interest

The authors declare no conflicts of interest.

## Data Availability

The data that support the findings of this study are available from the corresponding author upon reasonable request.

## References

[fsn372378-bib-0002] Al‐Farsi, M. , A. Al‐Amri , A. Al‐Hadhrami , and S. Al‐Belushi . 2018. “Color, Flavonoids, Phenolics and Antioxidants of Omani Honey.” Heliyon 4, no. 10: e00874. 10.1016/j.heliyon.2018.e00874. PMC6236019 30465028

[fsn372378-bib-0004] Araújo, F. G. D. , N. O. D. Araújo , V. R. L. D. Oliveira , R. H. D. L. Leite , F. K. G. D. Santos , and E. M. M. Aroucha . 2023. “Physicochemical and Bioactive Properties of Apis and Stingless Bee (Meliponini) Honey From Brazilian Caatinga.” Acta Scientiarum Animal Sciences 45: e59799. 10.4025/actascianimsci.v45i1.59799.

[fsn372378-bib-0006] Ares, G. , and S. R. Jaeger . 2023. “Check‐All‐That‐Apply (CATA) Questions With Consumers in Practice: Experimental Considerations and Impact on Outcome.” In Rapid Sensory Profiling Techniques, 257–280. Woodhead Publishing. 10.1016/B978-0-12-821936-2.00013-3.

[fsn372378-bib-0007] Arnaud, A. F. , R. A. da Silva , L. L. dos Santos Araújo , R. J. dos Santos Júnior , and D. A. de Oliveira Junior . 2008. “Perfil sensorial de méis de *Apis mellifera* L., 1758, Hymenoptera, apidae, produzidos na microrregião de Catolé do Rocha, PB.” Revista Verde de Agroecologia e Desenvolvimento Sustentável 3, no. 4: 15.

[fsn372378-bib-0008] Asih, N. E. , K. P. Ramadhanty , J. Ramandias , F. Azkarama , and W. B. Sunarharum . 2021. “Lexicon, Sensory Wheel and Kit as Sensory Communication Tools: A Review.” In IOP Conference Series: Earth and Environmental Science, vol. 924, no. 1, 012027. IOP Publishing. 10.1088/1755-1315/924/1/012027.

[fsn372378-bib-0064] Barth, O. M. 1989. Pollen in Brazilian Honey, 150. Oswaldo Cruz Institute.

[fsn372378-bib-0010] Bertelsen, A. S. , Y. Zeng , L. A. Mielby , Y. X. Sun , D. V. Byrne , and U. Kidmose . 2021. “Cross‐Modal Effect of Vanilla Aroma on Sweetness of Different Sweeteners Among Chinese and Danish Consumers.” Food Quality and Preference 87: 104036. 10.1016/j.foodqual.2020.104036.

[fsn372378-bib-0011] Borges, L. P. , and V. A. Amorim . 2020. “Metabólitos Secundários de Plantas Secondary Plant Metabolites.” Revista Agrotecnologia 11: 54–67.

[fsn372378-bib-0012] Bruneau, E. , E. Barbier , L. M. Gallez , and C. Guyot‐Declerck . 2000. “La roue des arômes des miels.” Abeilles et Cie (Belgium), (77). https://agris.fao.org/search/en/providers/122397/records/6472355353aa8c89630238fb.

[fsn372378-bib-0014] Carvalho, J. M. , F. L. Guedes , L. M. Carvalho , D. V. Costa Filho , and M. S. Madruga . 2026. “Chemical Screening, Botanical Origin, and Antioxidant Potential of *Apis mellifera* Honeys From the Brazilian Caatinga Biome.” Chemistry & Biodiversity 23, no. 4: e71184. 10.1002/cbdv.71184. 41983532

[fsn372378-bib-0017] Ciappini, M. , M. Vitelleschi , and A. Calviño . 2016. “Chemometrics Classification of Argentine Clover and Eucalyptus Honeys According to Palynological, Physicochemical, and Sensory Properties.” International Journal of Food Properties 19, no. 1: 111–123. 10.1080/10942912.2015.1020436.

[fsn372378-bib-0018] da Costa, A. C. V. , J. M. B. Sousa , T. K. A. Bezerra , et al. 2018. “Volatile Profile of Monofloral Honeys Produced in Brazilian Semiarid Region by Stingless Bees and Key Volatile Compounds.” LWT 94: 198–207. 10.1016/j.lwt.2018.04.043.

[fsn372378-bib-0019] da Costa, A. C. V. , J. M. B. Sousa , M. A. A. P. da Silva , D. dos Santos Garruti , and M. S. Madruga . 2018. “Sensory and Volatile Profiles of Monofloral Honeys Produced by Native Stingless Bees of the Brazilian Semiarid Region.” Food Research International 105: 110–120. 10.1016/j.foodres.2017.10.043. 29433198

[fsn372378-bib-0066] De Pelsmaeker, S. , G. De Clercq , X. Gellynck , and J. J. Schouteten . 2019. “Development of a Sensory Wheel and Lexicon for Chocolate.” Food Research International 116: 1183–1191. 10.1016/j.foodres.2018.09.063 30716904

[fsn372378-bib-0021] De Sousa, J. M. B. , E. L. de Souza , G. Marques , et al. 2016. “Sugar Profile, Physicochemical and Sensory Aspects of Monofloral Honeys Produced by Different Stingless Bee Species in Brazilian Semiarid Region.” LWT 65: 645–651. 10.1016/j.lwt.2015.08.058.

[fsn372378-bib-0022] Dobre, I. , L. A. Georgescu , P. Alexe , O. Escuredo , and M. C. Seijo . 2012. “Rheological Behavior of Different Honey Types From Romania.” Food Research International 49, no. 1: 126–132. 10.1016/j.foodres.2012.08.009.

[fsn372378-bib-0023] Escuredo, O. , I. Dobre , M. Fernández‐González , and M. C. Seijo . 2014. “Contribution of Botanical Origin and Sugar Composition of Honeys on the Crystallization Phenomenon.” Food Chemistry 149: 84–90. 10.1016/j.foodchem.2013.10.097. 24295680

[fsn372378-bib-0024] Feldmann, C. , and U. Hamm . 2015. “Consumers' Perceptions and Preferences for Local Food: A Review.” Food Quality and Preference 40: 152–164. 10.1016/j.foodqual.2014.09.014.

[fsn372378-bib-0025] Gunaratne, T. M. , C. G. Viejo , S. Fuentes , et al. 2019. “Development of Emotion Lexicons to Describe Chocolate Using the Check‐All‐That‐Apply (CATA) Methodology Across Asian and Western Groups.” Food Research International 115: 526–534. 10.1016/j.foodres.2018.10.001. 30599974

[fsn372378-bib-0026] Hofmanová, J. K. , J. Mason , and H. K. Batchelor . 2020. “Sensory Attributes of Coated Tablets: Developing a Formal Lexicon and Sensory Wheel.” International Journal of Pharmaceutics 590: 119883. 10.1016/j.ijpharm.2020.119883. 32946978

[fsn372378-bib-0027] IBGE . 2023. Instituto Brasileiro de Geografia e Estatística. Censo Agropecuário.

[fsn372378-bib-0028] Jaeger, S. R. , S. L. Chheang , D. Jin , C. M. Roigard , and G. Ares . 2020. “Check‐All‐That‐Apply (CATA) Questions: Sensory Term Citation Frequency Reflects Rated Term Intensity and Applicability.” Food Quality and Preference 86: 103986. 10.1016/j.foodqual.2020.103986.

[fsn372378-bib-0031] Koch, I. S. , M. Muller , E. Joubert , M. Van der Rijst , and T. Næs . 2012. “Sensory Characterization of Rooibos Tea and the Development of a Rooibos Sensory Wheel and Lexicon.” Food Research International 46, no. 1: 217–228. 10.1016/j.foodres.2011.11.028.

[fsn372378-bib-0032] Kortesniemi, M. , S. Rosenvald , O. Laaksonen , et al. 2018. “Sensory and Chemical Profiles of Finnish Honeys of Different Botanical Origins and Consumer Preferences.” Food Chemistry 246: 351–359. 10.1016/j.foodchem.2017.10.069. 29291860

[fsn372378-bib-0035] Lawless, H. T. , and H. Heymann . 2010. Sensory Evaluation of Food: Principles and Practices. Springer Science & Business Media.

[fsn372378-bib-0036] Lazaridou, A. , C. G. Biliaderis , N. Bacandritsos , and A. G. Sabatini . 2004. “Composition, Thermal and Rheological Behaviour of Selected Greek Honeys.” Journal of Food Engineering 64, no. 1: 9–21. 10.1016/j.jfoodeng.2003.09.007.

[fsn372378-bib-0037] Li, N. , M. Song , H. Li , et al. 2024. “Advancement of Foodomics Techniques for Honey Botanical Origins Authentication: Past Decade (2013–2023) and Future Perspectives.” Trends in Food Science & Technology 147: 104458. 10.1016/j.tifs.2024.104458.

[fsn372378-bib-0038] Liu, J. , Y. Li , B. Kassas , and T. Blare . 2025. “The Role of Consumer Awareness and Prior Exposure in Preferences for a New Regional Food Label: Evidence From Southwest Florida.” Food Quality and Preference 133: 105637. 10.1016/j.foodqual.2025.105637.

[fsn372378-bib-0040] Mahmoud, M. A. , Ö. Kılıç‐Büyükkurt , M. M. A. Fotouh , and S. Selli . 2024. “Aroma Active Compounds of Honey: Analysis With GC‐MS, GC‐O, and Molecular Sensory Techniques.” Journal of Food Composition and Analysis 134: 106545. 10.1016/j.jfca.2024.106545.

[fsn372378-bib-0041] Manoko, M. L. K. , and C. A. Mduda . 2024. “Harvesting Location Influences the Physicochemical Properties, Microbiological Quality and Sensory Attributes of Honey Produced by an Afrotropical Stingless Bee, *Axestotrigona ferruginea* .” Food and Humanity 3: 100433. 10.1016/j.foohum.2024.100433.

[fsn372378-bib-0042] Mongi, R. J. , and C. C. Ruhembe . 2024. “Sugar Profile and Sensory Properties of Honey From Different Geographical Zones and Botanical Origins in Tanzania.” Heliyon 10, no. 18: e38094. 10.1016/j.heliyon.2024.e38094. PMC11425160 39328532

[fsn372378-bib-0065] Moreira, F. I. N. , L. L. de Medeiros , L. M. de Carvalho , et al. 2023. “Quality of Brazilian Stingless Bee Honeys: *Cephalotrigona capitata*/mombucão and *Melipona scutellaris* Latrelle/uruçu.” Food Chemistry 404: 134306. 10.1016/j.foodchem.2022.134306 36283315

[fsn372378-bib-0044] Oroian, M. 2013. “Measurement, Prediction and Correlation of Density, Viscosity, Surface Tension and Ultrasonic Velocity of Different Honey Types at Different Temperatures.” Journal of Food Engineering 119, no. 1: 167–172. 10.1016/j.jfoodeng.2013.05.029.

[fsn372378-bib-0045] Oroian, M. , S. Amariei , I. Escriche , and G. Gutt . 2013. “A Viscoelastic Model for Honeys Using the Time–Temperature Superposition Principle (TTSP).” Food and Bioprocess Technology 6, no. 9: 2251–2260. 10.1007/s11947-012-0893-7.

[fsn372378-bib-0046] Ouchemoukh, S. , P. Schweitzer , M. B. Bey , H. Djoudad‐Kadji , and H. Louaileche . 2010. “HPLC Sugar Profiles of Algerian Honeys.” Food Chemistry 121, no. 2: 561–568. 10.1016/j.foodchem.2009.12.047.

[fsn372378-bib-0047] Piana, M. L. , L. P. Oddo , A. Bentabol , E. Bruneau , S. Bogdanov , and C. G. Declerck . 2004. “Sensory Analysis Applied to Honey: State of the Art.” Apidologie 35, no. Suppl. 1: S26–S37. 10.1051/apido:2004048.

[fsn372378-bib-0048] Roldán, A. , G. C. J. Van Muiswinkel , C. Lasanta , V. Palacios , and I. Caro . 2011. “Influence of Pollen Addition on Mead Elaboration: Physicochemical and Sensory Characteristics.” Food Chemistry 126, no. 2: 574–582. 10.1016/j.foodchem.2010.11.045.

[fsn372378-bib-0051] Santos, A. , R. F. A. Moreira , and C. A. B. De Maria . 2015. “Study of the Principal Constituents of Tropical Angico (*Anadenanthera* sp.) Honey From the Atlantic Forest.” Food Chemistry 171: 421–425. 10.1016/j.foodchem.2014.09.017. 25308689

[fsn372378-bib-0052] Seisonen, S. , E. Kivima , and K. Vene . 2015. “Characterisation of the Aroma Profiles of Different Honeys and Corresponding Flowers Using Solid‐Phase Microextraction and Gas Chromatography–Mass Spectrometry/Olfactometry.” Food Chemistry 169: 34–40. 10.1016/j.foodchem.2014.07.125. 25236195

[fsn372378-bib-0053] Siegmund, B. , K. Urdl , A. Jurek , and E. Leitner . 2017. ““More Than Honey”: Investigation on Volatiles From Monovarietal Honeys Using New Analytical and Sensory Approaches.” Journal of Agricultural and Food Chemistry 66, no. 10: 2432–2442. 10.1021/acs.jafc.6b05009. 28166628

[fsn372378-bib-0054] Smanalieva, J. , and B. Senge . 2009. “Analytical and Rheological Investigations Into Selected Unifloral German Honey.” European Food Research and Technology 229, no. 1: 107–113. 10.1007/s00217-009-1031-2.

[fsn372378-bib-0055] Špánik, N. J. O. V. I. 2014. “Identification of Volatile Organic Compounds in Honeydew Honeys Using Comprehensive Gas Chromatography.” Journal of Food and Nutrition Research 53, no. 4: 353–362.

[fsn372378-bib-0056] Tahir, H. E. , Z. Xiaobo , H. Xiaowei , S. Jiyong , and A. A. Mariod . 2016. “Discrimination of Honeys Using Colorimetric Sensor Arrays, Sensory Analysis and Gas Chromatography Techniques.” Food Chemistry 206: 37–43. 10.1016/j.foodchem.2016.03.032. 27041295

[fsn372378-bib-0057] Terrab, A. , A. G. González , M. J. Díez , and F. J. Heredia . 2003. “Mineral Content and Electrical Conductivity of the Honeys Produced in Northwest Morocco and Their Contribution to the Characterisation of Unifloral Honeys.” Journal of the Science of Food and Agriculture 83, no. 7: 637–643. 10.1002/jsfa.1341.

[fsn372378-bib-0058] Wei, Z. , J. Wang , and Y. Wang . 2010. “Classification of Monofloral Honeys From Different Floral Origins and Geographical Origins Based on Rheometer.” Journal of Food Engineering 96, no. 3: 469–479. 10.1016/j.jfoodeng.2009.08.028.

[fsn372378-bib-0059] Witczak, M. , L. Juszczak , and D. Gałkowska . 2011. “Non‐Newtonian Behaviour of Heather Honey.” Journal of Food Engineering 104, no. 4: 532–537. 10.1016/j.jfoodeng.2011.01.013.

[fsn372378-bib-0060] Xia, Y. , J. Hou , J. Chen , et al. 2025. “An Exploratory Study on the Development of a Sensory Wheel Affiliated With the Emotional Lexicon for Chrysanthemum Infusion.” Food Research International 201: 115428. 10.1016/j.foodres.2024.115428. 39849683

